# Observations on the Causes and Treatment of Ulcerous Diseases of the Leg

**Published:** 1835-10-01

**Authors:** 


### OBSERVATIONS ON THE CAUSES AND TREATMENT OF ULCEROUS DISEASES OF THE LEG. By *J. C. Spender*, Member of the Royal College of Surgeons in London. Octavo, pp. 210. London, 1835.

IT may seem to require no little confidence to write—it certainly demands considerable courage to read, a volume of upwards of two hundred pages on ulcers of the leg, in the year eighteen hundred and thirty-five. The disease is tolerably common—the treatment is not usually considered very difficult—and novelty would appear, from the nature of the subject, to be all but unattainable.

Yet Mr. Spender, a surgeon of Bath, has not been dismayed at the barrenness of the prospect, nor disgusted at the trifling nature of the subject.

He has witnessed in the last five years "about a thousand cases," and experience so vast has not been unproductive of excellent results. The present volume has sprung from the cases, a monument of the industry and the success of Mr. Spender.

The Preface, for there is a preface of eleven pages, contains an apology for writing:—

"Even if his opinions be found deficient in novelty, they are most of them original, so far as the Author is concerned. About five years ago, though ignorant, as he has since found, of some modes of treatment recommended, yet, acquainted with a number sufficient to be perplexed by their want of agreement, and the absence of any principle to hold them together, he commenced the task of observing for himself. Cases soon crowding upon his notice, afforded him the opportunity of drawing some conclusions; and his experience enlarging as he advanced, he was enabled to test their correctness by further observations and trials. A few months since, conceiving that the general and uniform nature of his practical inferences were such as authorized him to consider them, though not exactly in the light of established rules, yet as considerable approximations to the truth, he proceeded to write them down; intentionally abstaining from the examination of any treatise on the subject, until he had recorded the lessons his experience had taught him. In some instances his opinions have coincided with the statements of others; but, in a greater number, the doctrines and practice inculcated by those writers possessing the highest authority and reputation, were at such variance with his conclusions, as to determine him to publish his own." vi.

We shall see, or rather we may tell our readers, that Mr. Spender does not think his book altogether wanting in the quality of novelty. If he did, he would not, as he naïvely observes, be induced to publish it. He flatters himself, in fact, that his readers "will find some new truths struck out, and some old truths placed in a stronger and wider light; together with some errors, both new and old, examined and exposed. He believes, also, though a few may be slower to acknowledge than to perceive this, that the great bulk of readers are very fair and candid in their judgments."

Such are the anticipations of Mr. Spender, such are the objects with which he has communicated to the public and the profession the results of his extensive observations and mature reflections. That those observations and reflections are of value, must be evident from the concluding paragraph of the Preface.

"Whatever may be said or become of the Work which the Author thus ventures to publish, nothing can shake his confidence in the correctness of his opinions, which an increasing experience is daily confirming; and nothing shall rob him of the gratification he feels, in being able to describe a method of treatment, which, in his own hands, has already removed the sufferings of so many around him." xi.

This promise of novelty will stimulate the curious, and the prospect of utility will gratify the man of a practical turn of mind. We pass therefore with interest to the pages of the work. This contains four chapters. The first is devoted to—Remarks on the Causes and Classification of Ulcers of the Leg: the second is dedicated to the General Principles of Treatment; the third displays the Application of General Principles; and in the fourth are exhibited the Propriety and Safety of Healing Ulcers of the Leg. We fear we shall be compelled to hurry over the speculations and even the general reasonings of our author, in order to select the more practical portions for notice or for information. Yet the first chapter must, on some accounts, detain us.

Our author starts by recording and by combating the prevalent idea, that ulcers are more frequent and more obstinate in the lower than the upper limbs, on account of the dependent position of the former. This is not incidentally touched on, but the demolition of this vulgar error is made a cheval-de-bataille by Mr. Spender—is, in fact, brought prominently forward.

At the present time of day, the uprooting of a prejudice is so manifest a triumph, that it cannot be permitted to slip by us unobserved. We shall, therefore give a portion of the argument employed by Mr. Spender.

"The legs suffer from ulcerous diseases much more frequently than other parts of the body. This has been commonly explained by referring to their dependent position, or to their remoteness from the centre of the circulation: but that these circumstances, either separate or in combination, cannot account for the fact, must, I think, appear evident from the following considerations. The direction and locality of the limbs are perfectly natural conditions, and, therefore, to think that these lead to the production of mischief, seems to imply some original defect in their construction. There is, consequently, this presumption against the truth of both suppositions, that they necessarily drive us to the absurd conclusion, that the tendency of the structures is to evil. Without, however, insisting on this, the daily occurrence of facts affords a sufficient refutation of the opinions now under review. One individual has a spontaneous ulcer on his extremity, but for every such instance, perhaps twenty others never experience a similar sore during the whole of their lives. Two persons, of the same age, and of the same general health, shall receive a kick or a blow of the same severity on the same part of their legs—in the one case, the injury will get well of itself, or even in opposition to injudicious treatment; whilst in the other, the greatest care and attention to the mere local accident will not prevent it from running into a troublesome sore. Now the legs of all these four classes of individuals are equally dependent and equally distant from the centre of the circulation, and, consequently, if one or both of these states rendered them liable to the evil, the effects ought to be the same in each case. It is plain, therefore, that we must search for some other cause to explain the fact to be accounted for; and this, in a great majority of the worst instances, as I shall soon attempt to prove, appears to arise from the existence of a positive and palpable unhealthiness of the superficial structures of the limb." 2.

Let us venture, for a moment, to examine Mr. Spender's syllogisms. He denies the correctness of the general opinion, that the legs are more prone than the superior portions of the body to ulcers, because they are dependent, and because they are more remote from the centre of the circulation. His grounds for the denial seem to be reducible to these :—1, That the direction and locality of the limbs are natural conditions, and to suppose that natural conditions lead to evil is absurd; 2dly, That all persons have their legs lower than their heads, but that not one in twenty has ever a spontaneous ulcer on the leg.

We are not ourselves inclined to criticism; but one who was disposed to examine these positions closely, might observe that they are not of a very logical character. The absurdity of supposing that natural conditions lead to evil, can be felt by that class of philosophers only, who agree with Mr. Square in the eternal fitness of things, and think it a mistake to suppose that the Creator intended either moral or physical evil to be felt. As the body is composed of various parts, and as all those parts are in some way or other liable to disease, and even to death, it does not appear quite ridiculous to imagine that some should be rather more so than the rest. There would seem to be no great primâ facie absurdity in this. Facts are perhaps fallacious, yet facts are in favour of that absurdity, so monstrous in the eyes of Mr. Spender. The curves of the great arteries are the parts of those vessels most exposed, by the laws of physics, to suffer from the impulse of the circulating blood; singularly enough, those are the parts of the arteries most liable to atheromatous deposites, to ulceration, and to aneurism. The left ventricle of the heart is the portion exposed to most exertion and the greatest wear and tear; that ventricle is more frequently the seat of disease, of hypertrophy and dilatation, than any other part of the organ. The laws of gravitation would render the return of blood from the hemorrhoidal veins, and from those of the lower limbs, more difficult than from the veins of the head and arms: as a singular coincidence, the veins of the former are more often varicose than those of the latter are; nay, the conformation of the female pelvis, and the natural process of parturition, would tend, still upon physical principles and by mechanical means, to increase the disposition to a varicose state in the veins to which we have alluded; such is unfortunately and oddly, perhaps, the fact. We need scarcely particularise any further instances of this description; they will readily occur to all pathologists, and, indeed, to all practical men. Is the reader contented to suppose them mere coincidences, because Mr. Spender deems it absurd to imagine that a natural condition leads to evil—or does he feel inclined to admit, with some, that the body was not meant, by its all-wise Creator, to control all physical agencies and tendencies, but to accommodate itself to them, when possible, to make use of them, and, even when opposed to them, to be opposed at the smallest practicable quantum of expence of vital influence? This latter class of thinkers will not discover more absurdity in the notion that the lower limbs are weaker than the upper, in consequence of their situation and relations, than they find in the melancholy and undoubted truth, that if an individual's heels are tripped up, he inevitably falls, by the operation of the laws of gravity, upon his nose. The physiologist of this school may perceive in the anatomical construction of the veins, and in the disposition of its valves, an arrangement for facilitating the return of the venous blood, but no possible means of doing away with the action of those laws which the same Creator has imprinted on all matter, the general laws of physics.

The second argument of Mr. Spender is drawn from the admitted fact, that although, in all persons, the legs are lower than most other parts of their bodies, yet ulcers of the legs are comparatively unfrequent. To understand the force of this ingenious reasoning, it must first be recollected, that ulcers do really occur upon the legs more frequently than on the trunk, the head, or the arms, and that some general reason for this must exist. Mr. Spender conceives that the reason cannot be, the dependent position of the limbs in question, because that dependency is common to all persons, whilst ulcers are, happily, the property of but a few. The critic may observe, that no one has contended that ulcers must necessarily form upon the legs, because the return of the blood from them is less easy than it is from the head or from the arms; but they have supposed that this condition is calculated to confer a liability to their occurrence. Let us put an analogical case.—Twenty persons pass through the Pontine Fens, and two of the twenty catch an ague. Mr. Spender, we suppose, would assert with confidence, that the air of the Fens could not have produced an intermittent fever in two, as eighteen others, exposed to the same malaria, had escaped; or let us take another, and a still more parallel instance. A great many ladies are annually brought to bed in Great Britain. A certain proportion get varicose veins of the lower extremities—a certain number also become affected with hæmorrhoids. Mr. Spender will, of course, deny that parturition could occasion either the varices or hæmorrhoids, as all British mothers have not suffered from them.

In point of fact, the dependent situation of the legs has never, so far as we know, been looked upon as more than a predisposing cause of ulcers. Other and more powerful agencies must operate to summon them into existence. Most surgeons are prejudiced in favour of the idea so vigorously assailed by Mr. Spender, and their practice seems to countenance their notions. For the horizontal posture recommended by their theory, is equally recommended by its actual results, and we fear that the obstinacy of the present chirurgical generation will prove too strong for the logic and the cases of our author.

It may seem to some, that we have laid too much upon the back of this small question—that we have unnecessarily gone into the argument. But we wished to present a specimen of the reasoning of Mr. Spender, and to shew our readers a sample of the materials of which his book is made. That sample is, we think, sufficient, and we shall not dwell any further on what, to practical persons, may seem unprofitable matters of theory.

The great object of the first chapter of Mr. Spender's work is to prove that a varicose condition of the veins is a frequent, indeed a very frequent, cause of ulcers of the leg. He proves, by quotations from the works of Benjamin Bell and Sir Everard Home, that his is a discovery upon his part. It may be very true, that no writer on surgery has explicitly pointed out the fact, though, perhaps, even this may admit of doubt. Yet we feel well assured that, whatever surgical writers may omit, surgical practitioners, at all events in London, are not altogether ignorant of the circumstance. There is not a pupil, certainly there is no dresser at a London hospital, to whom both the term varicose ulcer, and the thing, are not "common as blackberries." We can safely declare that, great as is the stress which Mr. Spender lays upon the fact, he has not made it one atom more apparent than it was to us as students. If the surgical profession are so ignorant upon the matter as Mr. Spender evidently thinks them, we can only say that we ourselves were ignorant of that ignorance. Perhaps Mr. S. is right.

We recollect reading an elaborate paper in some penny journal, the object of which was to divide the community into two great classes—those who bought umbrellas, and those who stole them. Mr. Spender has almost as universal an idea of the importance of varicose veins; he divides all ulcers into those which are varicose, and those which are not so.

"All ulcerous diseases of the leg appear to admit of a very easy and natural arrangement into general and local; and, accordingly, this has been adopted by almost every writer. The first, or constitutional class, is subdivided in conformity to the nature of the diseases from which the sores arise—as venereal, scrophulous, and the like. The subdivisions of the second or local class, instead of being derived, as is usually the case, from the mere external aspect of the ulcers, should, I think, be taken from the more important and permanent distinction, arising from the presence or absence of the varicose state. All sores of this class may, therefore, be arranged under two orders, determinable by the condition of the veins—first, varicose; second, non-varicose. Under each of these orders may be retained the species or distinctions arising from the superficial appearance and character of the ulcer, according to the threefold degree of action exhibited—irritable, simple, indolent. Thus we should have varicose irritable, varicose simple, and varicose indolent; and non-varicose irritable, non-varicose simple, and non-varicose indolent. The two general classes may be farther considered as separate or combined, according as the specific sore takes place on a varicose or non-varicose limb; giving, for instance, venereal varicose, venereal non-varicose—each possessing its threefold degree of activity; and so of the rest. The object of such a classification is to make the presence or absence of the varicose affection one of the leading foundations on which the whole is built; as being the element of the greatest practical importance. When I have observed this simple distinction in practice, I have in general found that it is comparatively of little consequence whether the superficial aspect of the ulcer be what is termed irritable or indolent—exuberant or callous. An attention to the varicose condition of the veins, when present, has, with nearly equal facility, removed each variety of ulcer, by the use of the same form of application hereafter described; whilst, on the contrary, the same application to the same kind of ulcer, so far as its mere external appearance is concerned, varies in its effects, just in proportion as it depends on the varicose state, unless great attention be directed to this constituent of the disease. Nothing can be clearer, therefore, that this last characteristic furnishes a much surer ground for a practical arrangement of ulcers than the former—or rather, that the former affords none at all." 53.

So we are presented with a simple arrangement of ulcers of the leg, founded on the presence or absence of varices. Suppose we have a scrofulous ulcer which becomes inflamed, or sloughs. All we have to do is to call it a varicose, or, haply, a non-varicose, scrofulous, inflamed ulcer, and the philosophical nomenclature is perfectly satisfactory. This is the sure ground for a practical arrangement of ulcers, which hitherto was not in existence. We were, indeed, in a state of Egyptian darkness before Mr. Spender's book made its appearance. That we are not exaggerating our hitherto unfortunate condition will be evident, we think, from the statement which Mr. Spender presents.

"Another practical evil resulting from the ordinary classification of ulcers, by their superficial appearance only, is the limitation which is given to the employment of pressure. The most strenuous advocates of the bandage and strapping appear very much to confine the use of these remedies to the description of sore, usually called indolent, and to prohibit their employment in what are denominated irritable ulcers. I have been long convinced from experience, that nothing can be more fallacious and unfortunate than this doctrine. What has always guided me in the application of compression is, not the condition of the ulcer, but the condition of the structures underneath; not the question whether the ulcer be indolent or not, but the question whether the veins be varicose or not. I shall in another place have occasion to shew that this deep-seated condition of the limb, is the leading circumstance which should direct us in the employment of pressure, and not the external aspect of the ulcerated surface. All that is here maintained is the fact, that the common arrangement of sores, tends to confine this most important agent within extremely too narrow bounds." 56.

It would appear from this, that whether an ulcer be indolent or irritable, whatever, in short, be the state of the sore, Mr. Spender disregards this minor consideration, and looks at the structures underneath. If this exposition of his practice be really correct, au pied de la lettre—if he treats all varicose ulcers as varicose, whether they be indolent, or irritable, or inflamed, then we must admit there is, indeed, some novelty in his opinions, some novelty, also, in his practice. But we shall see more completely, as we go on, whether such is the actual meaning of our author. The conclusion of the chapter exhibits him well satisfied with the speculative part of his discoveries.

"Instead, therefore, of a more complicated, and, I believe, sometimes, misguiding classification, I have been in the habit of practically arranging all kinds of ulcers under these two very general heads—as they possess, or as they want, the varicose affection and its consequences. A primary attention to this will be found greatly to simplify our views and management of these diseases: and nearly all that is required by the method of treatment hereafter described, with the exceptions there pointed out, is to observe this comprehensive division." 59.

Fifty-nine pages have been occupied with the task of proving, that varices of the lower limbs are not the result of their dependent posture, and that ulcers are frequently the consequence of varices. His first position may possibly be questioned by the prejudiced part of the surgical world—his second may be thought, by those who captiously deny all claims to novelty and invention, to be little more than what is familiarly known. Without presuming to offer an opinion, we proceed to the consideration of the second chapter. It is devoted to—

### The General Principles of Treatment.

Mr. Spender remarks, that two circumstances chiefly demand our attention in the management of ulcerous diseases of the legs—the superficial sore upon the surface, and the deep-seated affection of the veins and other structures underneath. Mr. S. divides his treatment into the observance of two leading principles—first, the imitation of a natural process; secondly, the introduction of a healthy action. Such, then, are the great doctrines enunciated by our author, and their truth is not diminished by the spirit of humility with which he exposes the absurdity of all, or most other modes of treatment but his own.

"Now," says he, "if we compare this beautiful, simple, and effective process (the natural process of healing wounds by a clot of blood, or by scabbing) with the method of treating wounds and ulcers in the human subject by poultices and fomentations, vapours and lotions, together with spongings, washings, and wipings, how striking the difference appears; and it is really wonderful that we can continue to go on pursuing a plan which so violates these first lessons of nature. We must become more humble as well as more observant, and be contented to abandon our complicated and cumbersome remedies, which, by losing their simplicity, not uncommonly lose their effect, before we can hope to make much advancement in the treatment of diseases. Nature is to be conquered only by submitting to her; and he conquers the most easily who submits the most readily." 63.

One would almost be tempted to imagine, that no such thing was practised as the endeavour to procure union by the first intention. Wounds in the human subject are treated, according to our author, by poultices, fomentations, and so forth. How this may be in Bath, we do not know; but certainly it is not so ordered in London. In this, as in the instance of varicose ulcers, Mr. Spender would seem to have imitated, in some measure, that animal which puts its head in a hole, and, seeing nothing, concludes there is nothing to be seen. But we must not lose sight of his principia. Well, Mr. Spender, in attacking Nature, takes care not to lose sight of his stoop-to-conquer tactics.

"In obeying this principle of imitation in the management of ulcerous diseases of the leg, I have been in the habit of attending to two things;—employing an application which will form an incrustation, to resemble in its effects the natural scab, and removing the dressings as seldom and with as little disturbance as possible." 64.

A scab, then, as Hamlet says, is "the thing," and the object is to procure it. Mr. Spender's method is this:—

"Of all the kinds of outward application which I have tried, an ointment containing a very large quantity of prepared chalk forms the best artificial crust. The earthy matter must be in a much greater proportion than enters into any ointment in the Pharmacopœia, consisting of about three pounds of chalk to two pounds of lard. Even four pounds of chalk will be readily taken up by two pounds of lard; and if about three ounces of olive oil be added, the ointment will not be too stiff, but will easily admit of being spread on the linen. The best method of preparing this application is not by rubbing the chalk down with the lard; but, having previously reduced the chalk to a very fine powder, heat the lard to a tolerable temperature, and, whilst it continues hot, gradually add the levigated chalk in the same vessel in which the lard was warmed. By this means it forms more of a solution than a mere addition; and the two ingredients thus become more intimately blended together. This should be stirred until it is nearly cold, and then placed by for use." 64.

The effect of the ointment is that an incrustation is formed, first on the surrounding skin, then on the margins, and at last on the face of the ulcer. This crust is produced in a very gradual manner; and when the discharge is great, sometimes none is deposited for the first two or three dressings. It is this slow method of its formation that constitutes its excellency; as in this respect it resembles the concretion of the natural scab. In short, the chalk ointment, prepared and employed after the manner of our author fulfils the two conditions required to imitate the natural process. It forms an artificial scab, and does not require such frequent disturbance to the sore as most other kinds of dressings.

Such is Mr. Spender's mode of treating the milder descriptions of sore. It is founded on the principle of covering the ulcer with a scab, under which cicatrization is effected undisturbed. Again we are inclined to ask if this is new—again we are almost led to inquire whether, to use a very homely proverb, there is not more noise than wool in all this. We have seen, in our time, a fair number of trials to heal sores under a scab. One gentleman has done this with an absorbent powder—another has effected it by means of gold-beater's skin—a third by the eschar produced by the lunar caustic—and now comes Mr. Spender with his new chalk ointment. All these methods have been successively brought forward as specifics in their way—each has been successively vaunted as the best—what Mr. Spender's will turn out to be we shall not venture to predict. In his hands it has of course been successful in an eminent degree.

When the veins are varicose, or adventitious deposits exist, or the tunics of the skin are altered in their texture, or the mechanical form of the sore is very uneven, or any specific poison is present, to produce its influence on the part;—the unassisted efforts of the system are not competent to establish the cure; and, consequently, the chalk ointment, or any other application which derives its benefit from a resemblance to the natural process, must be equally ineffective. Such is the admission of Mr. Spender, and, therefore, he proceeds to his second general principle of treatment—the introduction of a healthy action.

The circumstances which render an ulcer difficult to heal are, according to our author—1, Varicose veins—2, Adventitious deposites—3, The form of the ulcer—4, The existence of constitutional disturbance or debility. For the latter, of course, constitutional treatment is required—for the former there is one remedy, Mr. Spender's main stay, " powerful and well-adjusted compression of the limb." He occupies many pages in explaining the modus operandi of compression. That he thinks it capable of doing a great deal, might be guessed from his almost universal employment of it. That guess is, however, more than confirmed by the following remarks.

" I have generally found that the combined application of the two general principles, thus briefly explained, has fully succeeded in practice. The object which has always been kept in view is, to unite them both in such a manner, that the veins and other structures may be supported underneath, whilst the ulcer is properly protected above. When this is done, many ulcers, which at the first inspection appear to be very unhealthy, and would seem to demand some local remedy to cleanse and correct them, put on a more favourable aspect as fast as the pressure restores a more natural action in the parts below. Being satisfied that it is the diseased condition of the veins and other structures, whenever it exists, that exerts the chief influence on the character of the sore, and accounts for its obstinacy, primary attention is directed to this condition, and an improvement in the structures is soon followed by a corresponding improvement in the appearance of the ulcer. When this conversion from an unhealthy to a healthy state has taken place in the sore, the chalk ointment forms a protecting crust to shield its face, and a firm cicatrix is soon produced underneath its covering. Even, therefore, when an ulcer is very ill-conditioned at first, I do not begin by applying poultices to draw it or stimulants to correct it, but simply cover it with the chalk dressing, tightly apply the bandage, and wait and see if its character do not quickly alter under the use of the pressure. When this most useful remedy has acted in the manner already described, the aspect of the sore is almost always changed; and its correction being chiefly accomplished from within, I do not commonly attempt, in the beginning of the treatment, to effect it from without. I have found from experience, that a little patience at the commencement is amply compensated for in the progress of the treatment. For, in almost every case, as soon as the compression alters the condition of the subjacent structures, the character of the superficial ulcer improves, and heals much more readily under the chalky crust than by the use of external stimulants and correctives. The propriety of this method of proceeding I find more and more confirmed by every day's experience, and I now rarely or ever have recourse to any local or external corrective, how faulty and vitiated soever the sore may appear, until I have given the tight pressure and mild application a fair and proper trial." 87.

Mr. Spender, as our readers may now perceive, is not at all afraid of riding his hobby hard. Tight pressure and the mild application are applied, whatever the state of the sore may be. Great as is the confidence we feel inclined to place in his advice, we should fear tight bandaging in a case of inflamed or irritable ulcer. This may not square with Mr. Spender's theory; but it certainly does seem to be the fair conclusion, from what we have observed. With this observation we arrive at the third chapter—the application of general principles. That chapter contains one hundred pages, and we cannot but admire the multiplying process which can scatter so little matter as there is over so wide a surface.

Mr. Spender commences by a lengthened condemnation of the abuse of poultices, a condemnation which, however just, is certainly possessed of a very small amount of novelty. He attacks, in the next place, though not so violently, the practice of enjoining rest in the treatment of ulcers of the leg. He is almost disposed to think rest injurious.

"But (says he) I am satisfied experience will permit us to go farther still, and that it authorizes us to maintain that not only is confinement unnecessary and useless, but absolutely pernicious, by depriving us of a direct and positive advantage which exercise gives to our treatment. It is not enough to say that an ulcer can be healed, notwithstanding the limb is moved, but I believe the cure is hastened by allowing it to follow its ordinary uses, provided it be properly supported by the assistance of a tight bandage. Exercise of the limb, during the healing process, acts beneficially in two very different ways. In the first place, it is a stimulus to the action of the parts,—the small vessels, by this means, becoming more energetic in their functions, form more vigorous and healthy granulations, as well as produce them in a shorter period. In the second place, at the same time that the exercise, in combination with pressure, is producing this internal change in the formation and character of the granulations, it is acting as a check on their growth; and by this resistance indirectly increases their strength." 104.

The reasoning is certainly ingenious; but, so far as we have seen, it is ingenuity versus fact, for, in the very great majority of ulcers of the leg, that have fallen under our observation and management, the general result has been—the quieter the limb, the quicker the recovery. We fear that the prejudice in favour of repose will be removed with difficulty by Mr. Spender. Yet he speaks with confidence; for he observes that the healthy stimulus given by exercise to the granulations "explains one of the reasons why ulcers get well so *quickly* and permanently, whilst individuals are walking about." It is melancholy when Doctors disagree about facts, yet, on this point, we must disagree with Mr. Spender. He makes his patients walk about pretty briskly.

"From a persuasion that confinement is useless in the cure of ulcers on the leg, and from having seen this opinion verified in many instances, where patients have come to me after having obtained a temporary cure by lying in bed with their legs as bad as ever, I have entirely renounced the remedy, with the single exception where active inflammation exists on the limb. Instead of encouraging rest, even when the patient is able and inclined to observe it, I direct him to use the limb, if suffering from an indolent sore, to the full or even greater extent than that to which the individual has been accustomed; but if from an irritable one, to a degree rather less." 107.

The result has been, as our readers might anticipate, satisfactory in a high degree. The patients who walk recover as fast as those who are confined, and their cure is far more permanent. We must proceed, with our author, to dress our ulcer.

"When I am called to see a patient with a bad leg, I begin by throwing away the poultice, if he happen to have one on—and by telling him to get up, if he happen to be in bed. With the single exception, where the process of sloughing exists and is incompleted, the poultice is entirely abandoned; and unless active inflammation is present, the individual is directed not to lie in bed more than other people." 108.

The limb is now examined *very* attentively, to discover whether there are varices or not. That is, of course, to be determined with great care. The next step is to examine the condition of the integuments in general, and the ulcer in particular The skin surrounding the sore for some distance, or even separate portions of it, may be excoriated or partially ulcerated; and when this is the case, the chalk dressing must be applied so as to cover such parts, as well as the face of the sore itself. If incompleted sloughing exists in the ulcer, the poultice and fomentation may be continued a little longer. If active inflammation is going on, the ordinary antiphlogistic remedies must be employed, and comparative rest must be enforced. These are the sole exceptions allowed by Mr. S. to his general method of treatment. He smiles at the terms irritable and indolent, exuberant and callous; he dispenses with the farrago of washes and ointments, and proceeds in most cases, undisturbed by the superficial appearance of the ulcer, to apply his peculiar method.

"The first thing is, to cover the surface and sides of the sore for some distance beyond its edges with the chalk ointment, spread about the thickness of a wafer on thin linen. I think the linen is preferable to lint, as it seems to allow the disengagement of the chalk more readily. No compresses of any sort are placed on this, as I am convinced the frequent use of paddings of linen, calico, and the like, placed between the dressing and the roller, are prejudicial, by unnecessarily loading and heating the part, and by confining, or preventing the escape of the matter." 114.

The face of the sore and the surrounding integuments, for some little distance, as well as any other portions of the skin which happen to be excoriated, being covered with a thin linen dressing of the chalk ointment, the next stage of proceeding consists in the application of the bandage. Ten or twelve pages are occupied with a description of the manner in which a roller should be used. The directions may be advantageous to a gentleman in the first twelve months of his apprenticeship, but are certainly not required by any who have arrived at the completion of their studies. Yet one point ought not to be passed in total silence. It is the force which Mr. Spender employs. "With regard to the tightness (he says) with which the roller is applied, I use much more force than is generally employed, pulling it at each turning as strongly as I can by the common effort of the arm, and sometimes even to the full extent of power which I possess." We confess that this does alarm us to a slight degree. We have seen one or two unfortunate results from over-tight bandaging, and our author's roller seems to be *rather* tight.

The dressing and bandage having been put on, the next question is, when they should be taken off again. The interval must be regulated by the circumstances of the case. Mr. S. recommends that the dressings should be removed as seldom as possible. Our author is as particular in undressing as in dressing an ulcer.

"The bandage being undone, the thin linen dressing is raised very gently, in order to allow as much of the chalk ointment to be left behind as may be attached to the surrounding parts and surface of the sore. We shall frequently find, even after the first dressing, that a thin film is formed about the margin, especially at the upper side, however large the ulcer may be; and if it be a small one, in addition to the deposit on the edges, some of the incrustation may probably be observed on the face of the sore. *This must not by any means be taken off:* it is treason to touch the chalky scab. It is, I believe, with some surgeons, a very common habit in the treatment of ulcers to wash the bordering skin and edges of the sore, and sometimes to pour water over its centre at each time of dressing. No good, however, can possibly be obtained by this; whilst some evil, and much loss of time, must be the consequence." 137.

The chalky scab, at the circumference of the sore, increases in size as that heals, and gradually encroaches on its centre. During the progress of the cure, the chalky scab is not in general to be dislodged from the surrounding integuments; but when the sore is very extensive, and the healing is proportionally slow and tedious, the successive deposits on the bordering skin occasionally form so thick a crust as to require its removal. The reason why it is advisable sometimes to take off this is, because it produces a layer sufficiently high to prevent the ulcer itself from experiencing all the good effects from the use of the pressure; this thickness of the bordering parts preventing the uncovered centre of the sore from being compressed as much as it ought. When the ulcerated surface is gradually covered by the chalky crust, and its whole extent is included, it should not be disturbed until a sufficient time has been allowed for the entire part to heal underneath.

Such is the general, we might almost say universal, plan of treatment advised by Mr. Spender. There are, however, two exceptions—extremely deep and callous sores on the one hand, and highly irritable and specific sores on the other. Both of these varieties are improved and assisted by the use of stimulating applications to the ulcer, for the purpose of exciting a healthy action in the former, and for the purpose of altering a morbid action in the latter.

We believe we have given a fair and tolerably accurate account of the substance of Mr. Spender's work. To some, our notice may appear too long—to the author, we are sure it will seem too brief. Its pretensions are great—its execution must be left to the opinion of the public. Such works as these are not altogether undeserving of attention. If good, they should be encouraged—if bad, they should be repressed—the best way of effecting either object is, to shew what they really contain.

